# Research Trends in Chronic Pain Physiotherapy: A Bibliometric Analysis

**DOI:** 10.3390/healthcare14142034

**Published:** 2026-07-08

**Authors:** Tomasz Jurys, Mateusz Grajek

**Affiliations:** 1 Department of Rehabilitation, Faculty of Health Sciences in Katowice, Medical University of Silesia in Katowice, Medyków 12, 40751 Katowice, Poland; 2 Department of Public Health, Faculty of Public Health in Bytom, Medical University of Silesia in Katowice, Piekarska 18, 41902 Bytom, Poland; mgrajek@sum.edu.pl

**Keywords:** chronic pain, physical therapy modalities, rehabilitation, bibliometrics, patient-centered care, musculoskeletal pain

## Abstract

**Background/Objectives**: Chronic pain represents a major global health burden and significantly impacts quality of life and functional capacity. Physiotherapy plays a central role in its management, yet the rapid growth of research in this field makes it difficult to identify current trends and emerging directions. The aim of this study was to analyze global research trends in chronic pain physiotherapy using a bibliometric approach. **Methods**: A bibliometric analysis was conducted using data retrieved from PubMed, Scopus, and Web of Science. A predefined search strategy was applied to identify relevant publications. Data were analyzed and visualized using VOSviewer. Co-occurrence analysis of terms based on titles and abstracts was performed using full counting. The most relevant terms were selected using a relevance score threshold, and a thesaurus file was applied to improve data quality. **Results**: The number of publications increased steadily from 2015 to 2025, indicating growing research interest in chronic pain physiotherapy. Network analysis revealed key research clusters related to clinical interventions, functional assessment, and outcome evaluation. The most prominent and central terms included “pelvic pain”, “neck disability index”, and “radicular pain”. Overlay visualization identified emerging topics such as “stratified care”, “multidisciplinary rehabilitation”, and “psychometric property”, reflecting a shift toward personalized and patient-centered approaches. **Conclusions**: Research in chronic pain physiotherapy is rapidly expanding, with a clear transition from traditional intervention-focused approaches toward individualized, multidisciplinary, and outcome-driven strategies. These findings provide insight into current research directions and may support clinicians and researchers in identifying future priorities in chronic pain management.

## 1. Introduction

Chronic pain is recognized as a major global health problem affecting a substantial proportion of the population and representing a leading cause of disability worldwide [1,2]. It is associated with significant physical, psychological, and socioeconomic consequences, including reduced quality of life, impaired function, and increased healthcare utilization [3,4,5,6,7]. Unlike acute pain, chronic pain is now understood as a complex and multifactorial condition, often persisting beyond normal tissue healing and influenced by biological, psychological, and social factors [8,9].

Physiotherapy plays a central role in the management of chronic pain and includes a wide range of interventions such as exercise therapy, manual therapy, education, and behavioral approaches [10]. Contemporary physiotherapy interventions increasingly target not only physical impairments but also psychosocial contributors to pain, including fear-avoidance, catastrophizing, and altered pain perception [11,12]. Despite these advances, the field of chronic pain physiotherapy remains heterogeneous, with ongoing debates regarding the effectiveness of different treatment modalities and the optimal approach to patient management. For example, while exercise and education-based interventions are widely supported, the role of manual therapy and emerging techniques such as digital rehabilitation or virtual reality remains under investigation [13,14,15]. Furthermore, the growing volume of scientific literature makes it challenging to identify dominant research themes and emerging directions within the field.

Despite the growing body of research on chronic pain physiotherapy, the overall structure and evolution of this field remain insufficiently characterized. Traditional narrative and systematic reviews primarily focus on evaluating the effectiveness of specific interventions, often within narrowly defined clinical contexts. While these approaches provide valuable clinical insights, they do not offer a comprehensive overview of global research activity, thematic development, or emerging trends across the discipline [15,16].

In recent years, bibliometric analysis has emerged as a powerful method for quantitatively assessing scientific literature, allowing for the identification of research patterns, collaboration networks, and evolving topics within a given field [17,18]. Unlike conventional reviews, bibliometric approaches enable the visualization of large datasets and provide insights into the intellectual structure of research domains [19]. This is particularly relevant in rapidly expanding fields such as chronic pain physiotherapy, where the volume and diversity of publications make it challenging to identify key directions and knowledge gaps. To date, limited bibliometric studies have specifically focused on physiotherapy interventions in chronic pain, and existing analyses often address broader pain research without detailed consideration of rehabilitation-focused approaches [20]. As a result, there is a need for a comprehensive analysis that maps current research trends and identifies emerging topics within the physiotherapy domain.

Bibliometric analyses have become an established methodological approach for exploring the intellectual structure and evolution of scientific fields. Modern bibliometric studies combine performance analysis with science mapping techniques, including co-occurrence, co-citation, and bibliographic coupling, to identify thematic structures and research frontiers. Widely used software such as VOSviewer implements association-strength normalization and visualization algorithms specifically developed for bibliometric network analysis. Furthermore, previous methodological studies have emphasized that database selection substantially influences bibliometric outcomes because Web of Science, Scopus, and PubMed differ in journal coverage, indexing policies, and metadata completeness. Therefore, combining multiple databases may improve retrieval completeness, although it also requires careful harmonization and deduplication of records [21,22,23,24].

Although bibliometric studies have investigated broader areas of pain management, musculoskeletal disorders, and rehabilitation research, no recent study has specifically mapped the evolution of physiotherapy-oriented chronic pain research across multiple major bibliographic databases. Consequently, important questions remain regarding the intellectual structure of the field, the relationships between emerging topics, and the direction of current scientific development. Bibliometric analysis offers a complementary perspective to traditional reviews by identifying patterns of scientific production, thematic evolution, and research priorities that cannot be readily captured through conventional evidence syntheses. Therefore, the present study was designed not to evaluate treatment effectiveness, but rather to characterize the development of chronic pain physiotherapy research and identify emerging themes that may influence future scientific and clinical agendas.

Therefore, the aim of this study was to perform a bibliometric analysis of research trends in chronic pain physiotherapy. The study sought to identify major research clusters, evaluate the evolution of scientific output over time, and highlight emerging themes shaping the future of physiotherapy practice in chronic pain management.

## 2. Materials and Methods

### 2.1. Data Source and Search Strategy

The data used in this bibliometric analysis were retrieved from PubMed, Scopus, and Web of Science, widely used databases for biomedical and health-related research. A comprehensive search strategy was developed to identify relevant publications on chronic pain in physiotherapy. The search query combined terms related to chronic pain and physiotherapy, including synonyms and variations. The following search strategy was applied (example used for PubMed): *((“chronic pain”[All Fields]* OR *“persistent pain”[All Fields]* OR *“long-term pain”[All Fields])* AND *(“physiotherapy”[All Fields]* OR *“physical therapy”[All Fields]* OR *“rehabilitation”[All Fields]))* AND *((clinicalstudy[Filter]* OR *clinicaltrial[Filter]* OR *controlledclinicaltrial[Filter]* OR *guideline[Filter]* OR *meta-analysis[Filter]* OR *networkmetaanalysis[Filter]* OR *observationalstudy[Filter]* OR *randomizedcontrolledtrial[Filter]* OR *review[Filter]* OR *scopingreview[Filter]* OR *systematicreview[Filter])* AND *(english[Filter])* AND *(2015:2025[pdat]))*.

The searches were performed in March 2026. Records published between 1 January 2015 and 31 December 2025 were retrieved from all three databases. Because PubMed, Scopus, and Web of Science continuously update their indexing coverage, all searches were conducted during the same period to minimize temporal discrepancies between databases. Consequently, the analyzed dataset represents the status of database indexing as of March 2026 rather than the complete lifetime coverage of each database.

The study period (2015–2025) was intentionally selected to cover the most recent complete decade of scientific publications. Since data retrieval was performed in March 2026, the indexing of publications from 2026 was still ongoing and therefore could have introduced bias related to incomplete database coverage. Limiting the analysis to the last fully completed ten-year period ensured temporal comparability of publication counts and improved the reliability of trend analyses.

The Web of Science search was conducted within the Web of Science Core Collection, including the Science Citation Index Expanded (SCI-EXPANDED), Social Sciences Citation Index (SSCI), and Emerging Sources Citation Index (ESCI).

Records retrieved from PubMed, Scopus, and Web of Science were exported and merged into a single database. It should be acknowledged that identical publications may be assigned different document types and metadata across bibliographic databases. Previous methodological studies have demonstrated discrepancies in document classification, author affiliations, keywords, funding information, and citation metadata among Web of Science, Scopus, and PubMed. To minimize these inconsistencies, duplicate records were identified primarily using Digital Object Identifiers (DOIs). When DOI information was unavailable or inconsistent, duplicate detection was performed using combinations of article title, publication year, journal title, and author information. Following automated screening, all potential duplicates were manually reviewed to ensure dataset accuracy. Document type classification was verified after merging the datasets, and metadata conflicts were resolved using DOI information whenever available. When DOI data were unavailable, priority was given to metadata consistency across the article title, first author, publication year, and journal title. A detailed flowchart of study identification, screening, and inclusion is provided in the Supplementary Materials (Figure S1) [25,26].

For bibliometric mapping, term co-occurrence analysis was performed using titles and abstracts. A minimum occurrence threshold of 15 was applied. After threshold filtering, VOSviewer relevance scoring was used to retain the most informative terms and reduce noise associated with generic expressions. A manually developed thesaurus file was employed to merge synonymous terms (e.g., “physical therapy” and “physiotherapy”) and remove non-informative words that did not contribute to thematic interpretation. The thesaurus file contained manually verified synonym mappings, spelling variants, abbreviations, and plural/singular forms. General methodological terms (e.g., “study”, “result”, and “analysis”) were excluded because they do not contribute to thematic interpretation. This manual standardization procedure was performed before network construction to reduce semantic redundancy and improve the interpretability of the resulting bibliometric maps, following the recommendations of the VOSviewer developers.

Because PubMed, Scopus, and Web of Science partially index different journals and publication types, a relatively modest proportion of duplicate records was expected. Manual verification confirmed that the remaining records represented unique indexed publications rather than unresolved duplicates.

### 2.2. Eligibility Criteria

The inclusion and exclusion criteria were defined to ensure the relevance and quality of the analyzed publications. Studies were included if they met the following criteria:

(i) Focused on chronic pain or persistent pain;
(ii) Addressed physiotherapy, physical therapy, or rehabilitation interventions;
(iii) Were published as original research articles or review papers;
(iv) Were published between 2015 and 2025.

Studies were excluded if they:

(i) Were conference abstracts, editorials, letters, or commentaries;
(ii) Did not primarily focus on physiotherapy or rehabilitation;
(iii) Were unrelated to chronic pain conditions;
(iv) Contained insufficient bibliographic information for analysis.

No restrictions were applied regarding the country of origin of the publications.

### 2.3. Data Extraction and Bibliometric Analysis

Bibliographic data were extracted from the records retrieved from PubMed, Scopus, and Web of Science. The extracted information included publication year, title, abstract, and author affiliations when available. The data were exported in a format compatible with bibliometric analysis and subsequently prepared for further processing. Prior to analysis, the dataset was screened to remove incomplete or irrelevant records. In addition, textual data from titles and abstracts were used for co-occurrence analysis of terms. A data cleaning process was performed to improve the quality of the analysis, including the removal of non-informative terms and the unification of synonyms using a thesaurus file.

Bibliometric analysis was performed using VOSviewer (version 1.6.20, Centre for Science and Technology Studies, Leiden University, The Netherlands). VOSviewer applies association-strength normalization and specialized visualization algorithms specifically developed for bibliometric network analysis. Network clustering was performed using the unified mapping and clustering framework proposed by Waltman et al. [21,22]. The software was used to analyze and visualize relationships within the bibliographic dataset [27]. A co-occurrence analysis of terms was conducted based on titles and abstracts. Full counting was applied, meaning that each occurrence of a term was counted equally across all documents. A total of 14,382 terms were initially identified from titles and abstracts. After applying a minimum occurrence threshold of 15, 648 terms remained. The 60% most relevant terms, as determined by the VOSviewer relevance score algorithm, were retained for final visualization. The thesaurus file contained 84 entries used for synonym merging and term standardization.

To ensure the clarity and interpretability of the results, a minimum occurrence threshold was applied, and only the most relevant terms were included in the final analysis based on a relevance score calculated by the software. A thesaurus file was used to remove non-informative terms and to merge synonyms, thereby improving the quality and consistency of the analysis. The resulting network was used to identify major research clusters and key topics within the field of chronic pain physiotherapy.

Citation analysis was performed using citation counts retrieved from the Scopus database on 20 April 2026. Raw citation counts were reported without normalization for publication year; therefore, older publications had greater opportunity to accumulate citations than more recent studies.

### 2.4. Visualization and Mapping

The visualization of bibliometric networks was performed using VOSviewer. Network visualization was applied to illustrate the relationships between terms based on their co-occurrence within the dataset. In the generated maps, each node represented a term, and the size of the node reflected the frequency of its occurrence. The distance between nodes indicated the strength of the relationship between terms, while the thickness of the connecting lines represented the strength of co-occurrence links. Clusters of related terms were identified using the built-in clustering algorithm, allowing for the detection of major thematic areas within the field. Additionally, overlay visualization was used to analyze temporal trends in the literature. In this approach, colors were assigned to terms based on the average publication year, enabling the identification of emerging and recent research topics.

## 3. Results

### 3.1. Characteristics of the Dataset and Publication Trends

A total of 4096 publications (after removal duplicates) were identified and included in the final bibliometric analysis, covering the period from 2015 to 2025. The dataset comprised original research articles and review papers retrieved from PubMed, Scopus, and Web of Science. However, the use of selected databases (PubMed, Scopus, Web of Science) may have resulted in the exclusion of relevant studies indexed elsewhere.

**Figure 1:**
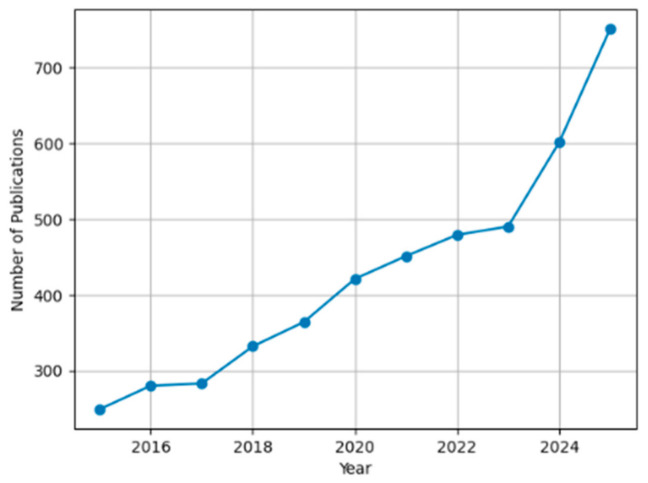
Trend of publications on chronic pain physiotherapy.

Over the analyzed period, the average number of publications was approximately 400–450 per year, indicating a substantial and consistent research output in the field of chronic pain physiotherapy (Figure 1). The dataset reflects a growing body of literature addressing various aspects of physiotherapy interventions, assessment methods, and multidisciplinary approaches to chronic pain management. These publications formed the basis for subsequent analyses, including trend evaluation, geographical distribution, and co-occurrence mapping of research topics.

The annual number of publications related to chronic pain physiotherapy demonstrated a consistent upward trend between 2015 and 2025 (Figure 1). The number of studies increased from 249 publications in 2015 to 751 in 2025, representing an overall growth of more than 200%. A gradual increase was observed between 2015 and 2018, followed by a more pronounced rise from 2019 onwards. In particular, the number of publications increased substantially after 2020, indicating an acceleration of research activity in recent years. The highest number of publications was recorded in 2025, highlighting the rapidly expanding interest in chronic pain physiotherapy. This trend suggests an increasing focus on evidence-based practice, the development of innovative therapeutic approaches, and the growing recognition of chronic pain as a major public health issue.

However, this increase should be interpreted cautiously. The observed increase in publication output should also be interpreted in the context of the continuous expansion of major bibliographic databases. Both Scopus and Web of Science have substantially broadened their journal coverage during the past decade, particularly through the inclusion of regional and emerging journals. Consequently, part of the observed publication growth may reflect improved database coverage rather than exclusively increased scientific productivity. Publication growth therefore likely reflects both genuine expansion of research activity and evolving database coverage [25,26].

### 3.2. Geographical Distribution

**Figure 2:**
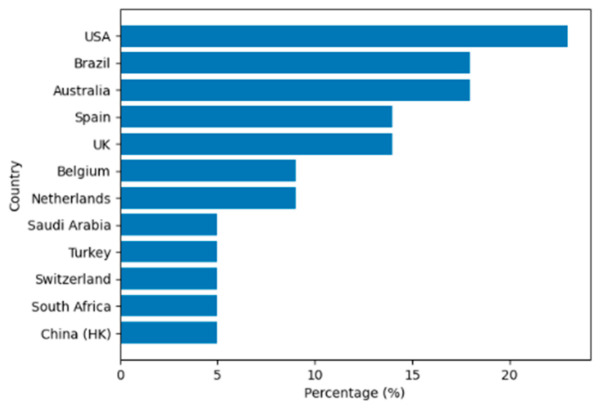
Distribution of publications by country (%).

The geographical distribution of publications was analyzed based on author affiliations, revealing a diverse global contribution to research in chronic pain physiotherapy (Figure 2). The geographical analysis should be interpreted cautiously because affiliation metadata are not fully standardized across bibliographic databases. Previous methodological studies have reported incomplete or inconsistent institutional addresses and country information, particularly for multi-author publications and older records. Therefore, country assignment in the present study reflects the indexed affiliation metadata available at the time of data retrieval rather than necessarily the complete institutional participation in each publication. The highest proportion of publications originated from the United States (23%), followed by Brazil (18%) and Australia (18%). European countries also contributed substantially, with the United Kingdom and Spain each accounting for 14% of publications, and Belgium and the Netherlands contributing 9% each. Other regions, including Saudi Arabia, Turkey, Switzerland, South Africa, and China (Hong Kong), accounted for smaller proportions of the total research output (approximately 5% each). Overall, the results indicate that research in chronic pain physiotherapy is predominantly concentrated in high-income countries with well-established research infrastructure. Notably, the significant contribution from Brazil highlights the growing role of emerging economies in the field of physiotherapy and pain research. These findings suggest an uneven global distribution of scientific output, which may reflect differences in research funding, healthcare systems, and the prioritization of chronic pain management across regions.

### 3.3. Bibliometric Mapping and Research Trends

**Figure 3:**
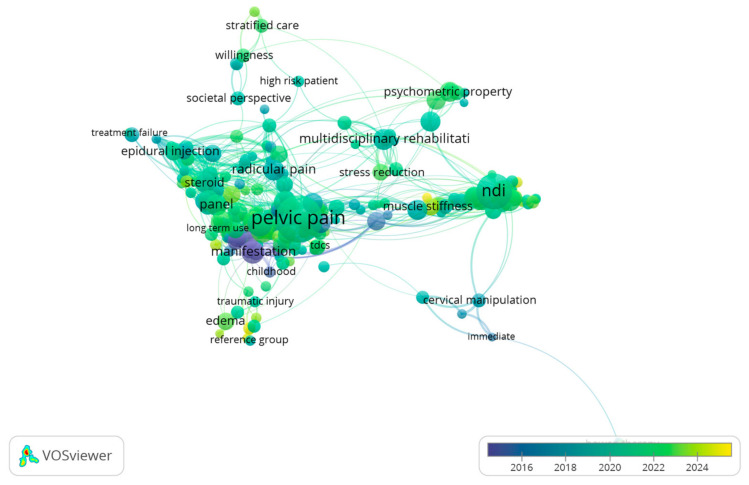
Co-occurrence network of terms and overlay visualization of research trends in chronic pain physiotherapy.

A co-occurrence analysis of terms based on titles and abstracts was conducted using VOSviewer, resulting in the identification of key research themes and their interrelationships within the field of chronic pain physiotherapy. The visualization of the bibliometric network is presented in Figure 3. In the generated network, each node represents a term, with node size corresponding to the frequency of occurrence. The proximity between nodes reflects the strength of the relationship between terms, while clusters of related terms indicate distinct thematic areas within the literature.

The analysis revealed several major research clusters. The first cluster was primarily associated with clinical and interventional approaches, including terms such as “epidural injection”, “steroid”, and other procedure-related concepts. This cluster reflects the integration of physiotherapy with medical and interventional pain management strategies. The second cluster was related to functional assessment and outcome measurement, with prominent terms including “neck disability index” and “muscle stiffness”. This cluster highlights the importance of objective evaluation tools in assessing treatment effectiveness and monitoring patient progress. A third cluster focused on modern and emerging approaches to chronic pain management, including “multidisciplinary rehabilitation”, “stratified care”, and “high-risk patient”. These terms indicate a growing emphasis on personalized treatment strategies and the identification of patient subgroups to optimize therapeutic outcomes. Another cluster encompassed pain mechanisms and clinical conditions, with terms such as “pelvic pain” and “radicular pain” representing commonly studied conditions within the field. These topics were strongly connected to both interventional and rehabilitation-based approaches, indicating their central role in chronic pain research. The analysis of term connectivity demonstrated that concepts such as “pelvic pain”, “neck disability index”, and “radicular pain” occupied central positions within the network, suggesting their strong associations with multiple research domains and their importance in the current scientific landscape.

Overlay visualization provided additional insights into temporal trends in the literature. More recent topics, indicated by warmer colors, included “stratified care”, “multidisciplinary rehabilitation”, and “psychometric property”. These emerging themes reflect a shift in research focus toward individualized, patient-centered care and the validation of outcome measures. In contrast, earlier research was more strongly associated with traditional interventional and biomechanical approaches, highlighting a transition in the field from technique-oriented treatments toward more comprehensive and integrative models of care.

Overall, the bibliometric mapping indicates that chronic pain physiotherapy research is evolving toward a more holistic and evidence-based paradigm, incorporating multidisciplinary collaboration, personalized treatment strategies, and a strong emphasis on outcome measurement and clinical relevance.

### 3.4. Most Cited Publications

An analysis of the most highly cited publications (dated 20 April 2026) revealed key influential works shaping the field of chronic pain physiotherapy (Table 1). The most cited article was a clinical practice guideline by Qaseem et al. (2017), with 2448 citations, highlighting the central role of evidence-based recommendations in guiding noninvasive management of low back pain [28].

Several of the top-cited publications were systematic reviews and guidelines focusing on nonpharmacological interventions, including exercise therapy, psychological approaches, and multidisciplinary rehabilitation. For instance, the systematic review by Chou et al. (2017), with 691 citations, reinforced the effectiveness of nonpharmacologic strategies such as exercise, mindfulness-based interventions, and manual therapy [29].

Notably, the inclusion of the work by Louw et al. (2016), with 566 citations, emphasizes the growing importance of pain neuroscience education, reflecting a paradigm shift toward biopsychosocial models of pain management [30]. Similarly, the guideline by Paice et al. (2016) highlights the need for comprehensive, multidisciplinary approaches, particularly in complex populations such as cancer survivors [31].

More recent contributions, such as the review by El-Tallawy et al. (2021), indicate a continued expansion of research into multimodal and integrative treatment strategies, combining pharmacological and non-pharmacological interventions [32].

Geographically, the majority of the most cited publications originated from the United States, underscoring its dominant role in shaping global research directions in chronic pain management. However, contributions from other regions, including the Middle East, reflect an increasing globalization of research efforts.

Overall, the analysis of highly cited publications confirms the trends identified in the bibliometric mapping, particularly the shift toward noninvasive, multidisciplinary, and patient-centered approaches in chronic pain physiotherapy.

## 4. Discussion

### 4.1. Principal Findings

The present bibliometric analysis provides a comprehensive overview of research trends in chronic pain physiotherapy over the past decade. The results demonstrate a substantial and continuous increase in scientific output, with more than a twofold growth in the number of publications between 2015 and 2025. This trend reflects the growing recognition of chronic pain as a major public health issue and highlights the increasing importance of physiotherapy in its management [33].

The bibliometric mapping identified several key research domains, including clinical interventions, functional assessment, and multidisciplinary rehabilitation. Notably, terms such as “pelvic pain”, “neck disability index”, and “radicular pain” occupied central positions within the network, indicating their strong relevance across multiple research areas. These findings are consistent with previous studies emphasizing the heterogeneity and multidimensional nature of chronic pain conditions [1,2,34]. Furthermore, the analysis of highly cited publications revealed that clinical guidelines and systematic reviews play a dominant role in shaping the field, emphasizing the importance of evidence-based practice in chronic pain management [28,29,30,31,32].

### 4.2. Shift Toward Biopsychosocial and Multidisciplinary Approaches

One of the most important findings of this study is the clear shift from traditional, intervention-focused models toward biopsychosocial and multidisciplinary approaches. Emerging terms identified through overlay visualization, such as “multidisciplinary rehabilitation”, “stratified care”, and “psychometric property”, indicate a growing emphasis on individualized and patient-centered care. These findings are in line with previous literature highlighting the limitations of purely biomedical models and supporting the adoption of a biopsychosocial framework in chronic pain management [8,34]. Increasing evidence suggests that psychological and social factors, such as fear-avoidance beliefs and pain catastrophizing, play a crucial role in the persistence of chronic pain and should be addressed in physiotherapy interventions [8,9,11]. The growing prominence of pain neuroscience education and cognitively informed therapies further supports this transition, as demonstrated in highly cited studies showing improvements in pain perception, disability, and patient understanding [10,13,30].

While the bibliometric findings indicate increasing scientific attention toward concepts such as multidisciplinary rehabilitation, stratified care, and patient-centered management, these observations should not be interpreted as direct evidence of their clinical effectiveness or widespread implementation. Bibliometric analyses quantify scientific activity and thematic prominence within the literature rather than therapeutic outcomes. Consequently, the identified trends primarily reflect evolving research priorities and conceptual developments within the field.

Several factors may explain the increasing visibility of these themes. First, growing recognition of chronic pain as a complex biopsychosocial condition has challenged traditional biomedical approaches and encouraged the development of integrated treatment models. Second, healthcare systems increasingly emphasize value-based care, individualized treatment planning, and outcome measurement, which likely contributes to the growing prominence of stratified care and psychometric evaluation. Third, expanding evidence supporting interdisciplinary management has stimulated research on collaborative rehabilitation models involving physiotherapists, physicians, psychologists, and other healthcare professionals.

Taken together, these developments suggest an ongoing conceptual transition in chronic pain physiotherapy research from intervention-centered investigations toward broader frameworks focused on patient complexity, clinical decision-making, and individualized rehabilitation pathways.

### 4.3. Emphasis on Physiotherapy-Based Interventions

The analysis of the most cited publications indicates that physiotherapy-based interventions, including exercise therapy, manual therapy, and education-oriented approaches, are central to current research in chronic pain management. Highly cited clinical guidelines and systematic reviews consistently highlight these strategies as key components of conservative treatment [28,29,30,31,32]. This is consistent with the scope of the present study, which focuses specifically on physiotherapy and rehabilitation approaches. The prominence of these interventions in both the bibliometric mapping and citation analysis reflects their fundamental role in contemporary chronic pain management. Furthermore, the increasing visibility of approaches such as pain neuroscience education and multidisciplinary rehabilitation suggests a shift toward more comprehensive and patient-centered physiotherapy models. These findings support the growing recognition of physiotherapy as a primary and essential component of chronic pain care [34].

Fibromyalgia represents another important area within chronic pain physiotherapy. As a complex chronic pain condition characterized by widespread pain, fatigue, and functional limitations, fibromyalgia has contributed substantially to the development of contemporary rehabilitation approaches. Research in this area has increasingly emphasized exercise-based interventions, pain neuroscience education, self-management strategies, and multidisciplinary rehabilitation, reflecting broader trends observed across the chronic pain literature.

### 4.4. Global Research Distribution and Inequalities

The geographical analysis revealed that research output is concentrated in high-income countries, particularly the United States, Australia, and European nations. This pattern is consistent with previous bibliometric studies, which have shown that scientific production is often closely linked to economic resources, research funding, and institutional infrastructure [35]. However, the notable contribution from other countries suggests a growing involvement of emerging economies in chronic pain research. Despite this progress, disparities in global research output remain evident, which may affect the generalizability of findings and the implementation of physiotherapy interventions across different healthcare systems.

Several factors may contribute to the observed geographical disparities in scientific output. Research productivity is strongly associated with the availability of funding, the presence of established academic and clinical research infrastructures, and access to international scientific networks. Countries with well-developed healthcare and research systems are generally better positioned to support large-scale rehabilitation studies and multidisciplinary chronic pain programs. Publication practices may also play an important role, as English-language journals remain the dominant platform for international dissemination of research findings. Consequently, valuable research conducted in non-English-speaking regions may be underrepresented in major bibliographic databases. In addition, differences in national health policies and strategic priorities may influence research activity, particularly in countries where chronic pain management and rehabilitation are recognized as important public health challenges. The substantial contribution observed from Brazil may reflect long-term investment in physiotherapy education, rehabilitation sciences, and international research collaboration, demonstrating that strong scientific productivity is not limited exclusively to the highest-income countries.

### 4.5. Underrepresented Areas in Chronic Pain Physiotherapy Research

Although the bibliometric network was dominated by themes related to multidisciplinary rehabilitation, functional assessment, and musculoskeletal pain conditions, several clinically important areas appeared less prominent. One example is fibromyalgia, which remains one of the most extensively studied chronic pain disorders and represents a major challenge for physiotherapy practice. Similarly, electrotherapy-based interventions did not emerge among the dominant thematic clusters despite ongoing investigation of modalities such as transcutaneous electrical nerve stimulation (TENS) and related approaches. Recent evidence suggests that electrotherapy may provide short-term benefits for selected patients when incorporated into comprehensive rehabilitation programs, particularly in conditions characterized by widespread pain and central sensitization. The relative underrepresentation of these topics within the bibliometric maps may reflect changes in research priorities rather than a lack of clinical relevance [36].

### 4.6. Clinical and Research Implications

The findings of this study have important implications for both clinical practice and future research. The observed shift toward personalized and multidisciplinary approaches highlights the need for physiotherapists to adopt more comprehensive treatment frameworks that integrate physical, psychological, and social dimensions of care [34,35]. Additionally, the increasing focus on outcome measurement and psychometric properties reflects a growing demand for standardized and validated assessment tools. This aligns with previous research emphasizing the importance of reliable outcome measures in improving clinical decision-making and research quality [15,16]. From a research perspective, the identification of emerging topics such as stratified care suggests promising directions for future investigation. Personalized treatment approaches tailored to specific patient subgroups may improve treatment effectiveness and optimize resource allocation [15].

### 4.7. Limitations and Future Directions

This study has several limitations. First, bibliometric analyses provide information regarding publication activity, thematic evolution, and citation patterns but do not assess the methodological quality, clinical effectiveness, or real-world implementation of the identified interventions. Consequently, the findings should be interpreted as indicators of scientific attention rather than evidence of therapeutic value.

Second, the co-occurrence analysis was based exclusively on titles and abstracts; certain concepts discussed only within the full text of publications may not have been captured. Consequently, thematic relationships identified in the bibliometric maps should be interpreted as representations of visible research discourse rather than complete conceptual content.

Third, despite the inclusion of three major bibliographic databases, relevant publications indexed elsewhere may not have been captured. An additional limitation relates to the search strategy itself. Although broad chronic pain terminology was used, specific diagnostic entities such as fibromyalgia were not explicitly included as independent search terms. Consequently, some condition-specific physiotherapy literature may have been underrepresented in the final dataset. Future bibliometric studies may benefit from incorporating major chronic pain diagnoses separately in order to provide a more comprehensive representation of the field.

Fourth, citation analyses are inherently influenced by publication age, journal visibility, and citation practices. Older articles generally have more opportunity to accumulate citations than recently published studies.

Finally, part of the observed increase in publication output may be attributable to the continuous expansion of database coverage and indexing practices in major bibliographic sources such as Scopus and Web of Science. Therefore, publication growth should not be interpreted exclusively as evidence of increased scientific productivity within the field.

Future research should aim to integrate bibliometric approaches with qualitative evidence synthesis to better understand the content and clinical relevance of research trends. This recommendation is consistent with recent methodological literature advocating the integration of bibliometric techniques with qualitative evidence synthesis and citation network analyses to improve the interpretation of scientific development [37]. In particular, stratified care models warrant further investigation because they aim to match treatment strategies to patient-specific characteristics and risk profiles. Multidisciplinary rehabilitation is also likely to remain a major research priority given the growing recognition of biological, psychological, and social determinants of chronic pain. Additionally, the increasing prominence of psychometric evaluation suggests a continuing need for robust outcome measures capable of capturing clinically meaningful changes in patient status. Together, these developments indicate that future chronic pain physiotherapy research will increasingly emphasize precision rehabilitation, individualized treatment pathways, and integrated models of care.

## 5. Conclusions

This bibliometric analysis provides a comprehensive overview of research trends in chronic pain physiotherapy over the past decade, demonstrating a substantial and continuous increase in scientific output. The findings indicate that current research is increasingly focused on physiotherapy-based, patient-centered approaches, with a strong emphasis on individualized treatment strategies, multidisciplinary rehabilitation, and outcome-oriented care.

The identification of key research clusters and emerging topics highlights a clear transition from traditional, technique-oriented interventions toward more integrated and biopsychosocial models of physiotherapy. In particular, the growing relevance of concepts such as stratified care and pain neuroscience education reflects the evolving complexity of chronic pain management.

These results underline the central role of physiotherapy in addressing chronic pain and provide valuable insights into the direction of future research. Further studies should focus on the clinical implementation and effectiveness of emerging approaches, particularly in diverse healthcare settings.

## Figures and Tables

**Table 1:** Top 5 most cited publications in chronic pain and physiotherapy-related research *.

No.	First Author	Year	Title	Journal	Citations	Country
1	Qaseem et al. [28]	2017	Noninvasive treatments for acute, subacute, and chronic low back pain: a clinical practice guideline from the American College of Physicians	Annals of Internal Medicine	2448	USA
2	Chou et al. [29]	2017	Nonpharmacologic therapies for low back pain: a systematic review for an American College of Physicians clinical practice guideline	Annals of Internal Medicine	691	USA
3	Louw et al. [30]	2016	The efficacy of pain neuroscience education on musculoskeletal pain: a systematic review of the literature	Physiotherapy Theory and Practice	566	USA
4	Paice et al. [31]	2016	Management of chronic pain in survivors of adult cancers: American Society of Clinical Oncology clinical practice guideline	Journal of Clinical Oncology	521	USA
5	El-Tallawy et al. [32]	2021	Management of musculoskeletal pain: an update with emphasis on chronic musculoskeletal pain	Pain and Therapy	370	Saudi Arabia/USA

* Citation counts were obtained from Scopus on 20 April 2026.

## Data Availability

The original contributions presented in this study are included in the article/Supplementary Materials. Further inquiries can be directed to the corresponding author.

## Supplementary Materials

The following supporting information can be downloaded at: https://www.mdpi.com/article/10.3390/healthcare14142034/s1.
