# Supplementary material for: Research Trends in Chronic Pain Physiotherapy: A Bibliometric Analysis
Source: Healthcare (Basel). 2026 Jul 8;14(14):2034. doi: 10.3390/healthcare14142034 (PMC13411719; doi:10.3390/healthcare14142034)
Supplement: Supplementary file 1 [file healthcare-14-02034-s001.zip › healthcare-4322014-supplementary.pdf]

Supplementary Materials

Research Trends in Chronic Pain Physiotherapy: A Bibliometric Analysis

Supplementary Material S1. Database Search Strategies

PubMed
((“chronic pain” OR “persistent pain” OR “long-term pain”) AND (“physiotherapy” OR “physical therapy” OR “rehabilitation”)) AND (Clinical Study OR Clinical Trial OR Controlled Clinical Trial OR Guideline OR Meta-Analysis OR Network Meta-Analysis OR Observational Study OR Randomized Controlled Trial OR Review OR Scoping Review OR Systematic Review) AND English language AND publication years 2015–2025.

Scopus
TITLE-ABS-KEY(("chronic pain" OR "persistent pain" OR "long-term pain") AND ("physiotherapy" OR "physical therapy" OR rehabilitation)) AND PUBYEAR > 2014 AND PUBYEAR < 2026 AND LANGUAGE(english) AND DOCTYPE(ar OR re).

Web of Science Core Collection (SCI-EXPANDED, SSCI, ESCI)
TS=(("chronic pain" OR "persistent pain" OR "long-term pain") AND ("physiotherapy" OR "physical therapy" OR rehabilitation)) Refined by: Document Types=(ARTICLE OR REVIEW) Languages=(ENGLISH) Timespan: 2015–2025.

Supplementary Methods S1. Deduplication Procedure

Records from PubMed, Scopus, and Web of Science were merged into a single dataset. Duplicate records were identified primarily using Digital Object Identifiers (DOIs). When DOI information was unavailable, duplicates were identified using article title, publication year, journal title, and author information. Potential duplicates were manually reviewed before final exclusion.

Supplementary Methods S2. Bibliometric Mapping Parameters

Bibliometric mapping was conducted using VOSviewer (version 1.6.20, Centre for Science and Technology Studies, Leiden University, The Netherlands). Full counting was applied. Terms were extracted from publication titles and abstracts. A minimum occurrence threshold was applied and the most relevant terms were selected using the VOSviewer relevance score algorithm. A manually developed thesaurus file was used to merge synonymous expressions and remove non-informative terms.

Supplementary Figure S1. PRISMA flow diagram of study identification, screening, eligibility assessment, and inclusion.

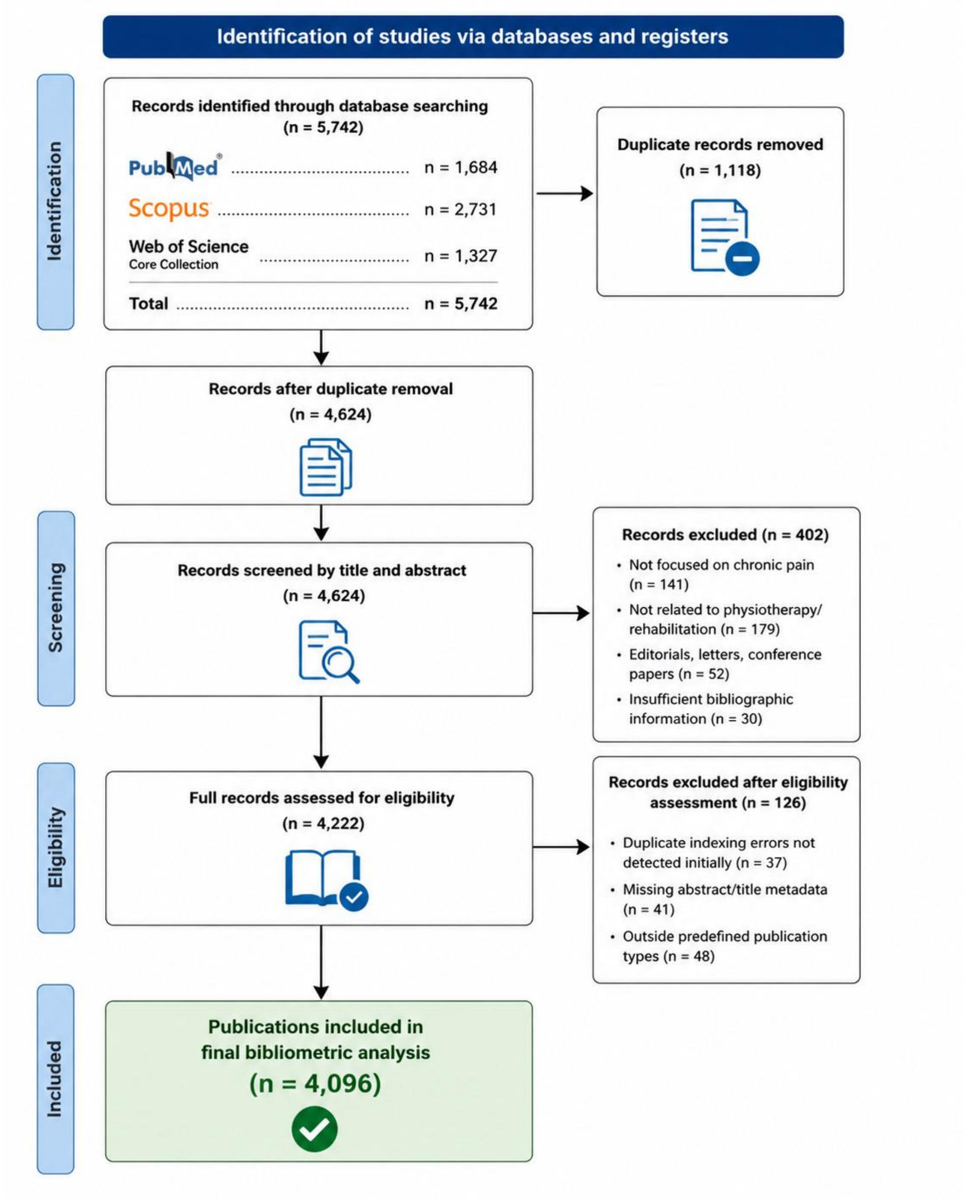

Figure S1. PRISMA flow diagram illustrating the identification, screening, eligibility assessment, and inclusion of publications in the bibliometric analysis of chronic pain physiotherapy research.
